# An AI-based segmentation and analysis pipeline for high-field MR monitoring of cerebral organoids

**DOI:** 10.1038/s41598-023-48343-7

**Published:** 2023-12-01

**Authors:** Luca Deininger, Sabine Jung-Klawitter, Ralf Mikut, Petra Richter, Manuel Fischer, Kianush Karimian-Jazi, Michael O. Breckwoldt, Martin Bendszus, Sabine Heiland, Jens Kleesiek, Thomas Opladen, Oya Kuseyri Hübschmann, Daniel Hübschmann, Daniel Schwarz

**Affiliations:** 1 Group for Automated Image and Data Analysis, Institute for Automation and Applied Informatics, Karlsruhe Institute of Technology, Eggenstein-Leopoldshafen, Germany; 2 Division of Pediatric Neurology and Metabolic Medicine, Department I, Center for Pediatric and Adolescent Medicine, Medical Faculty Heidelberg, Heidelberg University, Heidelberg, Germany; 3 Department of Neuroradiology, Heidelberg University Hospital, INF 400, Heidelberg, Germany; 4 Institute for Artificial Intelligence in Medicine (IKIM), University Hospital Essen, Essen, Germany; 5 German Cancer Consortium (DKTK), Heidelberg, Germany; 6 Cancer Research Center Cologne Essen (CCCE), Essen, Germany; 7 Computational Oncology Group, Molecular Precision Oncology Program, National Center for Tumor Diseases (NCT) Heidelberg, DKFZ, Heidelberg, Germany; 8 Pattern Recognition and Digital Medicine, Heidelberg Institute for Stem Cell Technology and Experimental Medicine (HI-STEM), Heidelberg, Germany

**Keywords:** Stem cells, Magnetic resonance imaging, Machine learning

## Abstract

Cerebral organoids recapitulate the structure and function of the developing human brain in vitro*,* offering a large potential for personalized therapeutic strategies. The enormous growth of this research area over the past decade with its capability for clinical translation makes a non-invasive, automated analysis pipeline of organoids highly desirable. This work presents a novel non-invasive approach to monitor and analyze cerebral organoids over time using high-field magnetic resonance imaging and state-of-the-art tools for automated image analysis. Three specific objectives are addressed, (I) organoid segmentation to investigate organoid development over time, (II) global cysticity classification and (III) local cyst segmentation for organoid quality assessment. We show that organoid growth can be monitored reliably over time and cystic and non-cystic organoids can be separated with high accuracy, with on par or better performance compared to state-of-the-art tools applied to brightfield imaging. Local cyst segmentation is feasible but could be further improved in the future. Overall, these results highlight the potential of the pipeline for clinical application to larger-scale comparative organoid analysis.

## Introduction

Cerebral organoids are key models to study human brain tissue and probe pathophysiological processes with tremendous potential for tailored therapeutic strategies. They are miniature 3D tissue cultures that are grown from human pluripotent stem cells. Cerebral organoids have been used to study neurodevelopmental disorders leading to microcephaly^1^ or neurological disorders like Alzheimer’s^2^ or Parkinson’s disease^3,4^.

The growing interest in organoid research over the past decade^5^ results in an increasing amount of data and thus calls for automated analysis and quantification. However, current automated organoid analysis pipelines are limited to smaller, e.g., intestinal, organoids^6^ or require organoid sacrifice^7^. Due to its non-invasive imaging technique, magnetic resonance imaging (MRI) allows for the generation of 3D time series of cerebral organoids at different developmental stages. Furthermore, brain MRI is the gold standard for diagnosis, staging, and treatment evaluation of various neurological disorders, thus highlighting its potential for imaging cerebral organoids, which has not yet been exploited. In comparison to brightfield imaging, which is the standard imaging technique for assessing organoid size and morphology^8–10^, MRI provides insight into 3D cerebral organoid morphology as well as functional tissue parameters through diffusion tensor imaging (DTI).

In the complex process of organoid cultivation, many routes of miss-differentiation of stem cells into other lineages can occur^8,11^. One important undesired route of organoid differentiation is marked by the occurrence of fluid-filled cavities (or ‘cysts’)^8,9^. Thus, accurately and automatically estimating organoid cysticity would greatly contribute to organoid quality monitoring. So far, however, only an approach for automated segmentation of exophytic cysts in patients with polycystic kidney disease using MRI has been reported^12^.

Here, we present the first application of MRI to human brain organoids using a neural network-based approach to extract cerebral organoid volume and structural features. Specifically, we address three crucial tasks for organoid monitoring and quality assessment: (I) organoid segmentation, (II) global cysticity classification, and (III) local cyst segmentation. Since brightfield imaging is the standard imaging technique for manual inspection of cerebral organoid size and cysticity^8–10^, we compare the performance of the automated analysis tasks for MRI and brightfield imaging.

## Results

### Organoid segmentation

**Figure 1:**
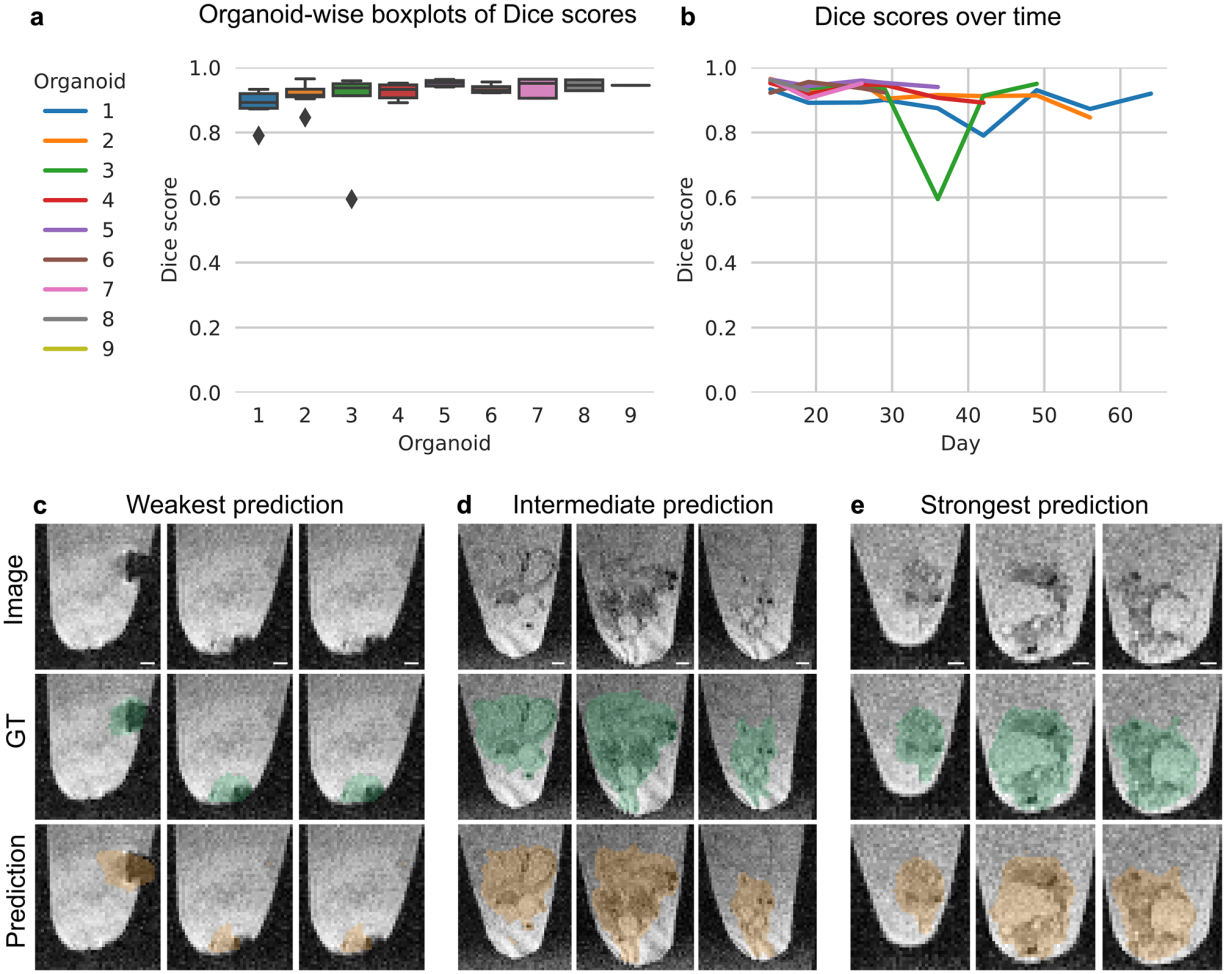
The 3D organoid segmentation in MRI. (**a**,**b**) Model performance. (**c**–**e**) Selected sagittal planes. (**c**) Organoid 3 (day 36): Dice score of 0.59. (**d**) Organoid 2 (day 42): Dice score of 0.91. (**e**) Organoid 5 (day 26): Dice score of 0.95. Image: original image, GT: Image with ground truth organoid location (green), Prediction: image with predicted organoid location (orange). Selected sagittal planes (left to right): (**c**) 50, 47, 44; (**d**) 58, 50, 40; (**e**) 52, 40, 34. For better visibility of the organoids and to investigate the detailed differences between ground truth and model prediction, we cut the images to the organoid location. Scale bar: 400 µm.

Organoid segmentation is essential to automatically extract features like organoid volume or structure. The state-of-the-art neural network SegFormer for 2D organoid segmentation applied to the standard imaging technique—brightfield imaging—achieved an overall Dice score of 0.91 ± 0.11 (mean ± SD, Supplementary Fig. 1). For organoid segmentation in 3D MRI images, the neural network 3D U-Net reached an overall Dice score of 0.92 ± 0.06 (mean ± SD; Fig. 1a,b). Even though the MRI model performs very accurately overall, we investigated challenging samples to identify the model’s weaknesses. The model performs poorest for Organoid 3 on day 36 (Dice score of 0.59). For this organoid, the disruption of one or more cystic structures resulted in a reduced overall volume (Supplementary Fig. 2) and a split of the organoid into multiple pieces (Fig. 1c). These pieces stick to the Eppendorf tube wall which causes that a part of the organoid border blurs with the MRI background. This biological outlier is unique in our dataset and was therefore difficult to be learned by the model. The analysis of other samples reveals a reliable detection by the model (Fig. 1d,e).

### Global cysticity classification

**Figure 2:**
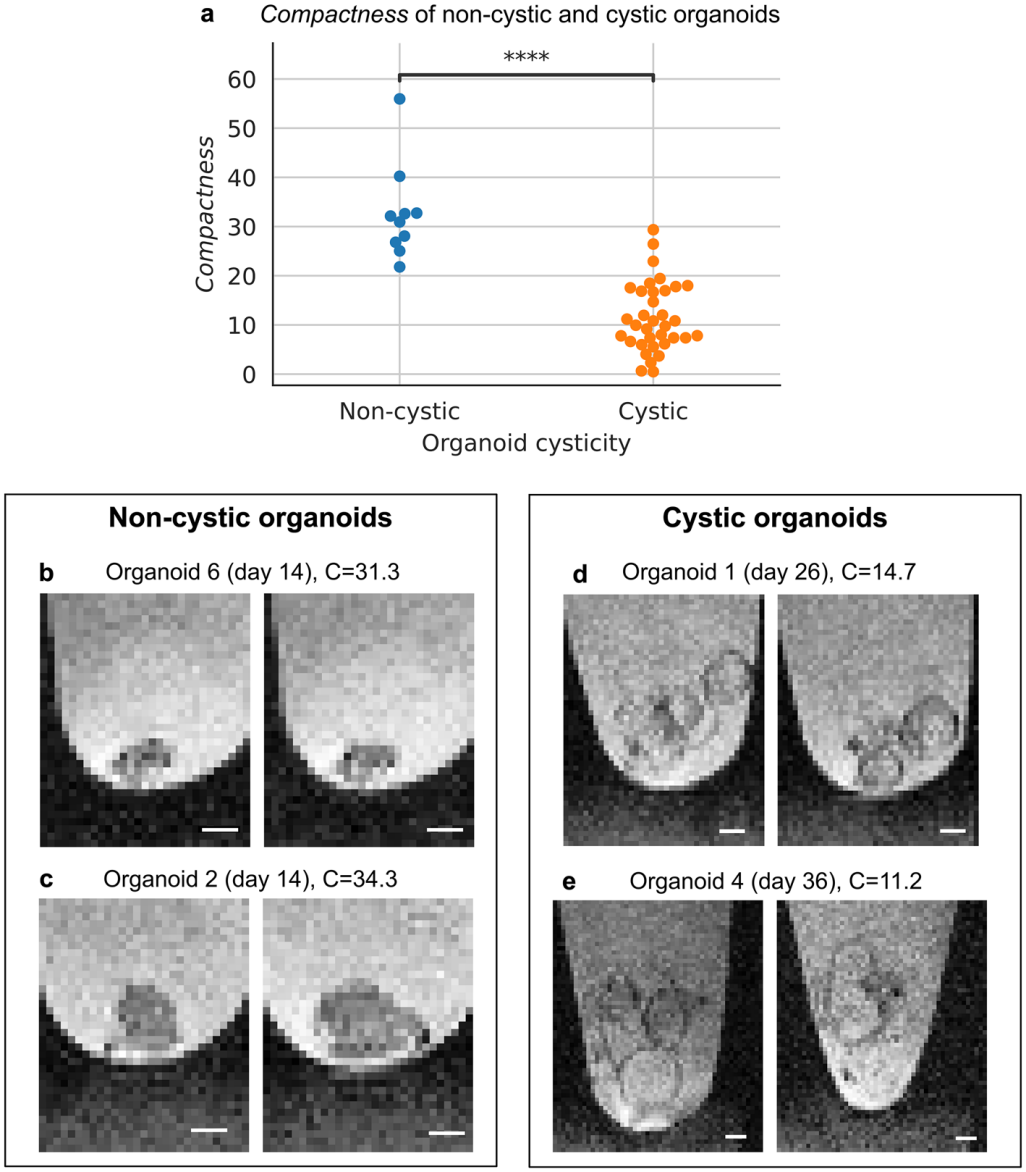
Global cysticity classification in MRI. (**a**) *Compactness* separates cystic and non-cystic organoids. ****P < 0.0001 two-sided t-test. (**b**–**e**) Selected sagittal planes from two cystic and two non-cystic organoids. Selected sagittal planes (left to right): (**b**) 59, 60; (**c**) 41, 45; (**d**) 58, 61; (**e**) 36, 53. For better visibility of the organoids, we cut the images to the organoid location. Scale bar: 400 µm.

Cyst formation is an undesired process during cerebral organoid cultivation^8,9^. Thus, accurately determining organoid cysticity can serve as a quality control tool. Qualitatively, non-cystic and cystic organoids show different morphologies in MRI and brightfield microscopy (Supplementary Fig. 3). For brightfield microscopy, the state-of-the-art neural network ResNet34, trained and evaluated for global cysticity classification, reached a Receiver Operator Characteristic area under the curve (ROC AUC) of 0.94 ± 0.08 (mean ± SD; Supplementary Fig. 5). For MRI, we developed the environment-based metric *compactness* which achieved a ROC AUC of 0.98 (Fig. 2), thus highlighting its role as a highly reliable quality control tool.

**Figure 3:**
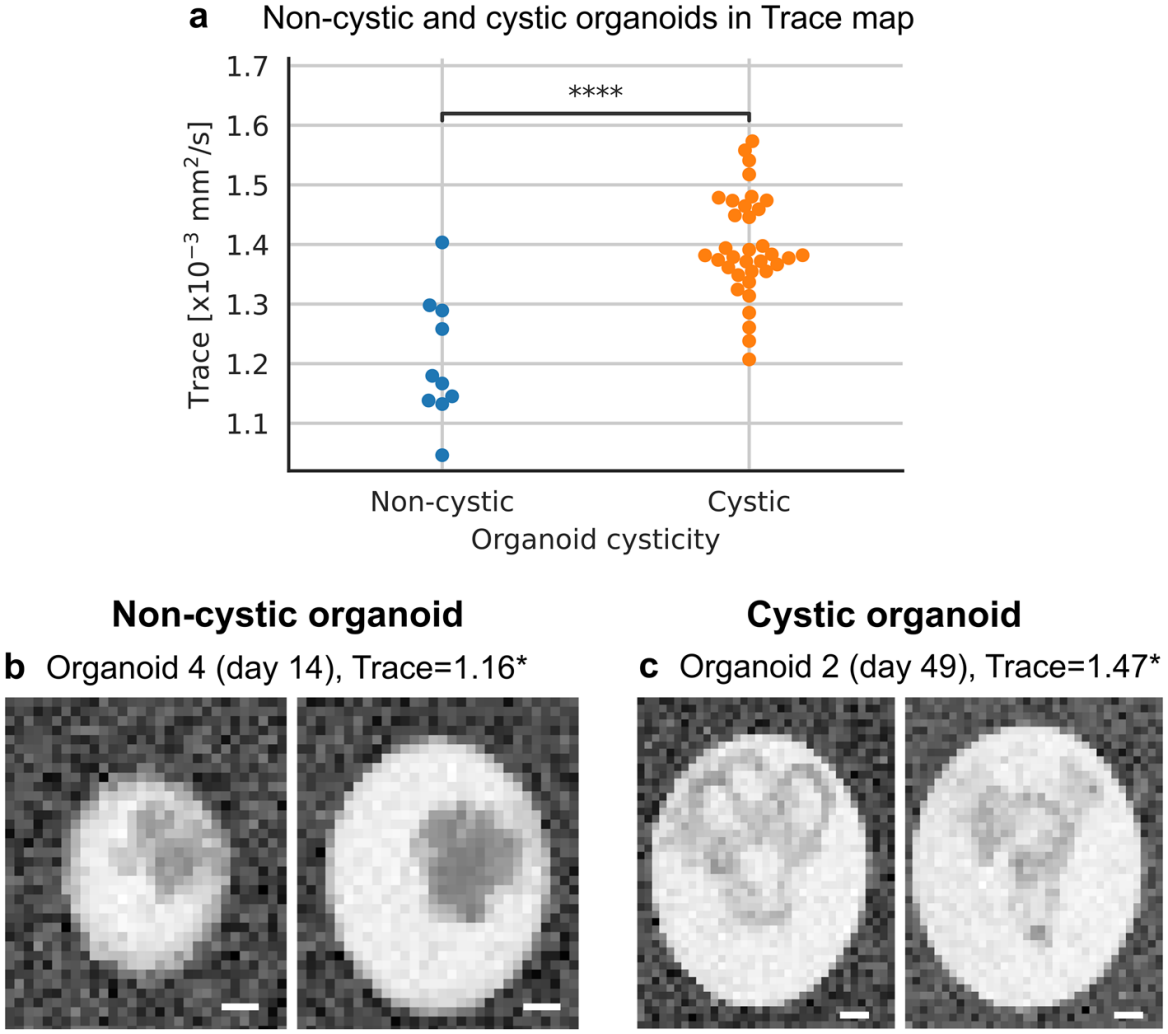
Diffusion tensor imaging (Trace map) shows different tissue characteristics of cystic and non-cystic organoids. (**a**) Trace of non-cystic and cystic organoids. ****P < 0.0001 two-sided t-test and Holm-Šídák correction to adjust for multiple testing of other DTI maps. (**b**,**c**) Selected coronal planes from one cystic and one non-cystic organoid; *[× 10^–3^ mm^2^/s]. Selected coronal planes (left to right): (**b**) 1, 2; (**c**) 5, 6. For better visibility, we cut the images to the Eppendorf tube boundaries. Scale bar: 400 µm.

Using diffusion tensor imaging (DTI), we observed that cystic organoids have a significantly higher average diffusion than non-cystic organoids (Fig. 3a). As can be seen in Fig. 3b,c, cysts have an increased diffusion compared to compact tissue. Analysis of other parameter maps are included in Supplementary Table 1.

### Local cyst segmentation

**Figure 4:**
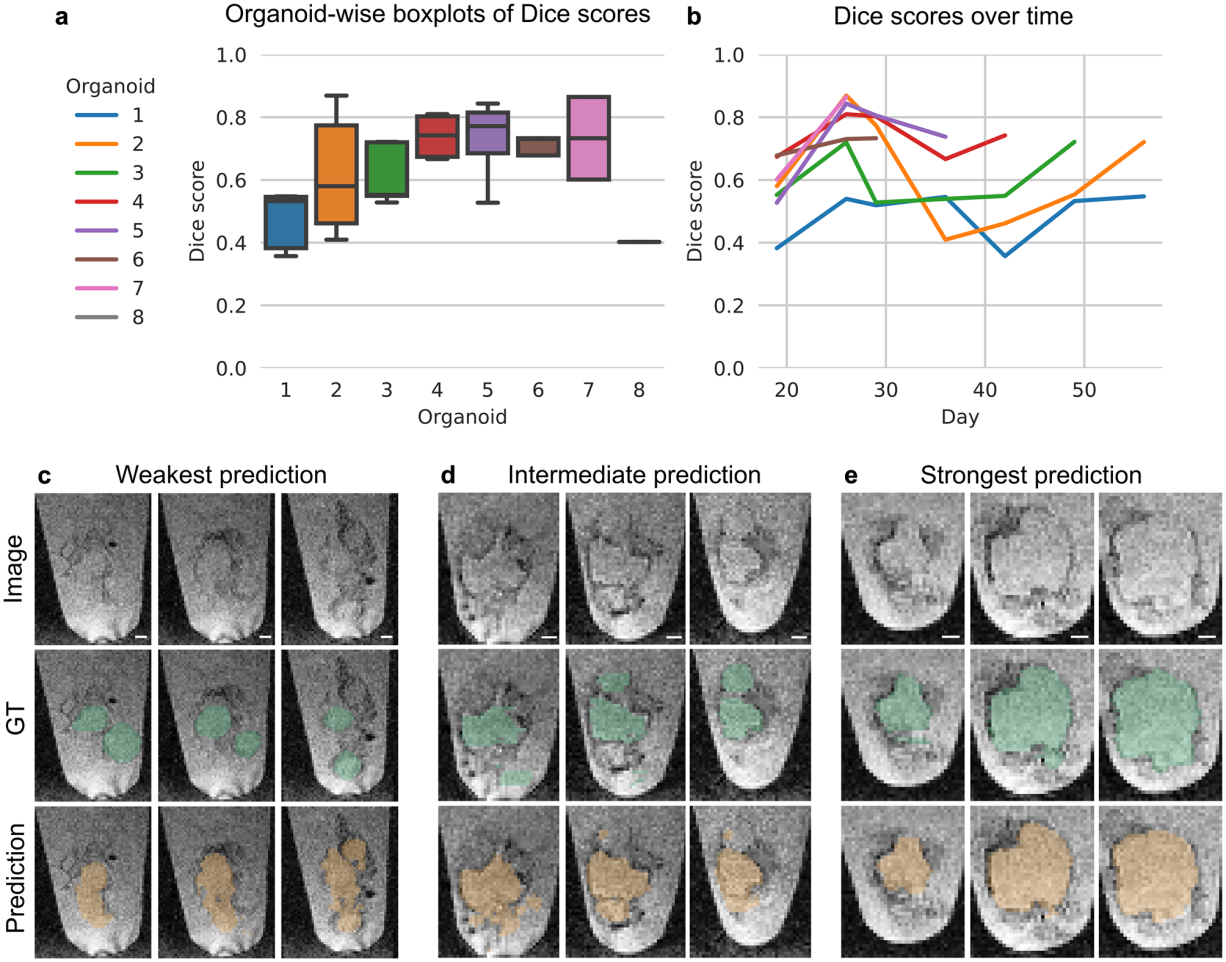
The 3D local cyst segmentation in MRI. (**a**,**b**) Model performance. (**c**–**e**) Selected sagittal planes for three organoids. (**c**) Organoid 1 (day 42): Dice score of 0.34. (**d**) Organoid 4 (day 36): Dice score of 0.63. (**e**) Organoid 7 (day 26): Dice score of 0.83. Image: original image, GT: image with ground truth organoid location (green), Prediction: image with predicted organoid location (orange). For better visibility of the organoids and to investigate the detailed differences between ground truth and model prediction, we cut the images to the organoid location. Selected sagittal planes (left to right): (**c**) 60, 55, 51; (**d**) 52, 49, 42; (**e**) 63, 56, 49. Scale bar: 400 µm.

The good performance for global cysticity classification raises the question of whether cysts can be segmented locally—which would provide further insight into cyst distribution and location. For this task, the 3D U-Net achieved an overall Dice score of 0.63 ± 0.15 (mean ± SD). As shown in Fig. 4a,b, the Dice scores for individual samples showed a large variation with values ranging from 0.34 to 0.83. The analysis of weak and intermediate model predictions showed discrepancies between model predictions and ground truth especially for organoids with many small cysts (Fig. 4c,d). The model performed especially well on images with large, clearly visible, and distinct cysts (Fig. 4e).

## Discussion

In this study, we introduce high-field MRI for the non-invasive monitoring and analysis of cerebral organoids using a neural network-based approach. Since neither thresholding nor using a 2D U-Net resulted in convincing results for MRI organoid segmentation (Supplementary Table 2), we used a 3D U-Net which achieved a mean Dice score of 0.92 for organoid segmentation. This is on par with AI-based organoid segmentation for brightfield imaging (mean Dice score of 0.91). Comparable methods for MRI brain segmentation achieve Dice scores in the range of 0.72 and 0.93^13–18^. Such a highly reliable automated analysis will represent a powerful tool to compare wild-type organoids with disease models associated with altered growth rate such as Zika-Virus disease^19^ or neurodevelopmental diseases leading to microcephaly^20^.

As the first step, reliable organoid segmentation paves the way for comprehensive, non-destructive quality monitoring including morphological and functional tissue parameters. For MRI, the newly introduced metric *compactness*, inspired by the concept of signal-to-signal ratio^21,22^, assesses overall cysticity. It successfully separated non-cystic and cystic organoids, closely matching the phenotypical appearance of previously reported non-cystic and cystic organoids (Supplementary Fig. 3, Supplementary Fig. 4)^8,9^, at an outstanding ROC AUC of 0.98 and outperformed the deep learning-based ResNet34 applied to brightfield imaging (ROC AUC 0.94). In contrast to brightfield imaging, which only provides morphological insight, DTI measures functional tissue parameters. Using DTI, it was shown that cystic organoids have a significantly higher diffusion than non-cystic organoids most likely reflecting higher fluid content. To further investigate cyst formation, especially using DTI, choroid plexus organoids could be an interesting model^9^. While our study primarily focused on detection of cystic miss-differentiation, our pipeline could likely also be applied to investigate necrotic core formation in larger, longer matured cerebral organoids which could be used as a quality control readout as well.

Successful global cysticity assessment led to the question of whether cysts can be segmented locally to differentiate solid compartments from fluid-filled cavities. While organoid segmentation and global cysticity classification were conducted in brightfield and MRI images for comparison, local cyst segmentation was performed for MRI images only. Due to the 2D nature of brightfield images, reliable annotation of cysts is rather difficult (Supplementary Fig. 3). Using the 3D MRI images, an annotation of cysts was more feasible, indicating the better assessment of the three-dimensional morphology using MRI images. The 3D U-Net trained for local cyst segmentation reached a mean Dice score of 0.63 which indicates a challenging segmentation task. Other challenging segmentation tasks such as ischemic stroke lesion segmentation achieve even lower Dice scores of 0.37 in MRI^23,24^ and 0.54 in CT^23,25^. Especially for organoids having many small cysts, correct local cyst segmentation appears to be a major challenge due to technical resolution and contrast-to-noise ratio limits. In such cases, global cysticity classification may thus capture more easily the fluent transition from compact to cystic organoids.

Some limitations need to be taken into consideration. On the one hand, reliable MRI organoid segmentation and global cysticity assessment could be achieved despite the relatively small dataset and heterogeneous organoid morphology with an equal or better performance compared to state-of-the-art AI-based methods applied to brightfield images. Thus, we do not expect a boost in performance here when extending the dataset. On the other hand, local cyst segmentation could probably benefit from a larger dataset. However, technical limitations of the image acquisition would most likely still impede segmentation performance in case of many small cysts due to uncertainty with respect to exact boundary detection for both human annotation and model prediction. Using standard plate formats for transfer-free high-throughput imaging is currently challenging due to the small NMR sample chamber and therefore requires to create tailored containers.

Overall, this work presents the first application of MRI for the non-invasive analysis of cerebral organoids. It was shown that cerebral organoids can be accurately monitored over time and for quality assessment using state-of-the-art tools for automated image analysis. In comparison to brightfield imaging, MRI gives better insight into 3D cerebral organoid morphology and DTI provides functional tissue characteristics of cerebral organoids. These results point out the potential of the pipeline for clinical application to larger-scale comparative organoid analysis.

## Materials and methods

The code to reproduce the results is publicly available on GitHub (https://github.com/deiluca/cerebral_organoid_quant_mri). All MRI images and annotations for organoid segmentation, global cysticity classification, and local cyst segmentation generated for this work are publicly available on Zenodo (https://zenodo.org/record/7805426).

### Differentiation of cerebral organoids

Organoids were generated according to^26^ with minor modifications. Wildtype iPSCs were singled and seeded at a density of 8 × 10^4^ cells/ml in a V-shaped 96 well plate in organoid formation medium (DMEM/F12, KnockOut Serum Replacement, NEAA, ß-mercaptoethanol) supplemented with 4 ng/ml bFGF (Peprotech) and Y-27632 (50 µM; StemCell Technologies) to induce embryoid body (EB) formation. The following day, the medium was exchanged to remove Y-27632 and lower the bFGF concentration to 2 ng/ml. On day 5, neural induction was initiated by exchanging the medium to neural induction medium (DMEM/F12, N2 supplement, NEAA, glutamine (all ThermoFisher), 1 µg/ml heparin (Sigma)) with a medium change on day 7. On day 9, EBs were embedded into Matrigel (Corning; growth factor reduced) droplets and cultivated until day 13 in organoid differentiation medium (ODM) 1 (DMEM/F12:Neurobasal medium 50:50, NEAA, glutamine, penicillin/streptomycin, N2 supplement, B27 supplement w/o vitamin A (all ThermoFisher), insulin (Sigma), ß-mercaptoethanol (ThermoFisher)). On day 13, organoids were excised from the droplets and transferred into a 12-well plate containing organoid differentiation medium 2 (DMEM/F12:Neurobasal medium 50:50, NEAA, glutamine, penicillin/streptomycin, N2 supplement, B27 supplement with vitamin A, insulin, ß-mercaptoethanol) and placed on a shaker in the incubator with medium exchange every 2–3 days. After imaging, the organoids were transferred back to the plate containing fresh medium and placed on the orbital shaker (Sunlab, 55 rounds/minute) for further development.

### Brightfield imaging

Brightfield images were taken using a Leica DMi1 microscope with included camera using LAS EZ software (Leica). Images were taken with a 10 × magnification from day 1 until day 9 of differentiation and were further followed with a 5 × magnification from day 12 until day 29.

### MRI

For MR measurements, organoids were transferred to 1.5 ml Eppendorf tubes containing standard ODM (T2-time of ~ 64 ms in this experimental setting) and conveyed to the MRI using warming packs for temperature control. In total, nine organoids were scanned at varying time points over a period of 64 days, resulting in 45 individual samples. Three Eppendorf tubes were placed next to each other in a holder, thus allowing simultaneous imaging of three organoids (Supplementary Fig. 6). Nine control organoids not undergoing MRI served as handling control. Before and after imaging, the medium was analyzed in both groups using a blood gas analyzer which showed that MRI had no specific negative effect on organoid health (Supplementary Table 3).

MRI was performed at room temperature using a high-field 9.4 Tesla horizontal bore small animal experimental NMR scanner (BioSpec 94/20 USR, Bruker BioSpin GmbH, Ettlingen, Germany) equipped with a four-channel phased-array surface receiver coil. Compared to common field MRI field strengths ranging from 1 to 7 Tesla, high-field 9.4 Tesla offers significantly improved spatial resolution and enhanced signal-to-noise ratio^27^. The MR protocol included the following sequences:

1. High-resolution T2*-weighted gradient echo sequence: 3D sequence, echo time (TE): 18 ms, repetition time (TR): 50 ms, 80 µm isotropic resolution, acquisition matrix: 400 × 188 × 100, flip angle: 12˚, number of averages: 1, duration: 15 min 40 s. This sequence was chosen to allow for accurate isotropic imaging and to account for potential susceptibility effects caused by e.g. neuromelanin^28^, cellular debris or calcifications.
2. DTI-spin echo sequence: 2D sequence, TE: 18.1 ms, TR: 1200 ms, 100 µm in-plane resolution, acquisition matrix: 120 × 50, slice thickness: 1.5 mm, number of diffusion gradient directions: 18 + 5 A0 images, b-values: 0/650 s/mm^2^, gradient duration: 2.5 ms, gradient separation: 15.5 ms, flip angle: 130°, number of averages: 1, duration: 23 min 05 s. This sequence was included to account for organoid inner structure including nerve fiber growth^29^.

### Organoid segmentation

Organoid segmentation was performed to assign each image voxel to one of two categories: organoid or non-organoid.

For MRI, we used min–max normalized images from the T2*-w sequence. Since simpler methods like Multi-Otsu’s threshold^30^ and a 2D U-Net^31^ did not deliver convincing results (Supplementary Table 2), we used a 3D U-Net^32^ for efficient (Supplementary Table 4) organoid segmentation. We trained the model with Adam (learning rate 1 × 10^−3^, weight decay 1 × 10^−7^) for 2000 iterations with batch size 1 and a weighted combination of binary cross entropy and Dice loss (1:10).

For brightfield imaging, Otsu’s threshold^33^ did not yield compelling results as well (Supplementary Table 5). Therefore, we used the SegFormer model^34^, implemented in^35^, for state-of-the-art 2D segmentation. We trained the model with Adam (learning rate 1 × 10^−4^, weight decay 1 × 10^−1^) for 2000 iterations with batch size 1 and a weighted combination of binary cross entropy and Dice loss (1:10). The config files for SegFormer training are provided on GitHub.

For evaluation of both models, we used the Dice score, which is commonly used to quantify the performance of image segmentation methods. It is defined as two times the area of the intersection divided by the total number of voxels in the ground truth and predicted segmentation (Eq. 1). A perfect segmentation corresponds to a Dice score of 1.

1 $$Dice\, score=\frac{2\cdot |A\cap B|}{|A|+|B|}$$

To get an unbiased estimate of the models’ performance, we used organoid-wise Leave-One-Out Cross-Validation (LOOCV). For each of the nine LOOCV splits, all images of one organoid are used for model testing, the remaining images of all other organoids are used for model training and model validation. For each LOOCV split, we used a random 80% training, 20% validation split for model selection. The Dice score in the Results section refers to the model performance on the LOOCV test set.

### Global cysticity classification

Global cysticity classification aims at determining the overall organoid cysticity: cystic (low-quality) or non-cystic (high-quality). To provide a reference ground truth based on the T2*-w sequence, an organoid was categorized as cystic if a cystic structure was detected within the organoid, corresponding to standard quality assessment on brightfield imaging (Supplementary Fig. 3) as previously reported^8,9^. Otherwise, it was categorized as non-cystic.

For automatic classification in MRI, we constructed the simple metric *compactness* which serves as an environment-based estimator of organoid cysticity (Eq. 2). It is based on the idea that cysts are filled with similar fluid like the medium under the assumption of relative B1-homogeneity in a stereotyped region close to the surface coil. Therefore, the more similar the organoid intensities are to the medium intensities, the more cystic the organoid is.

2 $$\begin{aligned}&Compactness: = abs\left[ {\upmu \left( {int_{{org}} } \right) - \upmu \left( {int_{{medium}} } \right)} \right] \hfill \\ &\mu \left( X \right): = \frac{1}{{\left| X \right|}}\sum _{{x \in X}} x \hfill \\ &abs(x) = \left\{ {\begin{array}{ll} {x\;,if\;x > 0} \\ { - x\;,otherwise} \\ \end{array} } \right. \hfill \\ &{\text{A}}{ \setminus }{\text{B}} = \{ {\text{x}} \in {\text{A}}:{\text{x}} \notin {\text{B}}\} \hfill \\ &int_{{org}} = \{ {\text{intensities}}\;{\text{of}}\;{\text{organoid}}\;{\text{voxels}}\} \hfill \\ &int_{{medium}} = \{ {\text{intensities}}\;{\text{of}}\;{\text{medium}}\;{\text{voxels}}\} \setminus int_{{org}} \hfill \\ \end{aligned}$$

While *int*_*org*_ was derived from the ground truth organoid segmentations, *int*_*medium*_ was determined by applying Otsu’s threshold^33^ 2D-wise along all organoid-containing coronal planes (Supplementary Fig. 7). The first and last organoid-containing coronal planes were discarded to filter artifacts caused by noisy medium intensities.

For brightfield imaging, concepts like *compactness* are not applicable due to heterogeneous microscopy backgrounds and the limited spatial visibility of cysts in the 2D image (Supplementary Fig. 3). Therefore, we used the state-of-the-art neural network ResNet34^36^ for binary classification, implemented in^37^. The model was trained with Adam (learning rate 1 × 10^−6^, weight decay 0) for 30 epochs with batch size 16 and a binary cross entropy loss. On-the-fly image augmentations included random rotation (0–360 degrees), random resized crop (scale 0.3–1.0, ratio 1.0) and ColorJitter (brightness = 0.1, saturation = 0.1, contrast = 0.1) to account for large variations in organoid size and color.

For the evaluation of *compactness* and the ResNet34, we used the area under the Receiver Operator Characteristic curve (ROC AUC). ROC AUC is a common metric for the evaluation of binary classification problems; a perfect classifier achieves a ROC AUC of 1. Since in contrast to *compactness*, the ResNet34 requires training, we used organoid-wise LOOCV for model evaluation.

To further probe tissue characteristics of cystic and non-cystic organoids, parameter maps (Trace; FA; 1st, 2nd_,_ and 3rd Eigenvalues) were extracted from the DTI sequence using the built-in analysis tool (Paravision 6.0, Bruker BioSpin GmbH, Ettlingen, Germany). We used a two-sided T-test to test for significantly different average diffusion and used Holm-Šídák to adjust for multiple testing.

### Local cyst segmentation

Local cyst segmentation aims at localizing cysts. For this task, we used the T2*-w sequence and manually annotated cysts. Due to the low-resolution images, especially smaller cysts are difficult to annotate. Therefore, we excluded organoids with less than 1000 voxels (0.51 mm^3^) in cysts and included 34 samples in total.

For segmentation, we trained and evaluated a 3D U-Net^32^ as for organoid segmentation but with 5000 training iterations. Local cyst segmentation was performed for MRI images only, because the annotation of cysts in brightfield images was not feasible due the limited spatial visibility of cysts in the 2D images (Supplementary Fig. 3).

### Supplementary Information

Supplementary Information.

## Data Availability

The datasets generated during this study are available at Zenodo (https://zenodo.org/record/7805426).
