# Supplementary material for: An AI-based segmentation and analysis pipeline for high-field MR monitoring of cerebral organoids
Source: Sci Rep. 2023 Dec 1;13:21231. doi: 10.1038/s41598-023-48343-7 (PMC10692072; doi:10.1038/s41598-023-48343-7)
Supplement: Supplementary file 1 — Supplementary Information. [file 41598_2023_48343_MOESM1_ESM.docx]

**An AI-based segmentation and analysis pipeline for high-field MR monitoring of cerebral organoids**

Luca Deininger*^†12^, Sabine Jung-Klawitter^†2^, Ralf Mikut^1^_,_ Petra Richter^2^, Manuel Fischer³, Kianush Karimian-Jazi³, Michael O. Breckwoldt³, Martin Bendszus³, Sabine Heiland³, Jens Kleesiek^456^, Thomas Opladen^2^, Oya Kuseyri Hübschmann^2^, Daniel Hübschmann^578^, Daniel Schwarz^3^

* luca.deininger@kit.edu

^†^ These authors contributed equally to this work

^1^ Group for Automated Image and Data Analysis, Institute for Automation and Applied Informatics, Karlsruhe Institute of Technology, Eggenstein-Leopoldshafen, Germany

^2^ Division of Pediatric Neurology and Metabolic Medicine, Department I, Center for Pediatric and Adolescent Medicine, Medical Faculty Heidelberg, Heidelberg University, Heidelberg, Germany

^3^ Department of Neuroradiology, Heidelberg University Hospital, INF 400, Heidelberg, Germany

^4^ Institute for Artificial Intelligence in Medicine (IKIM), University Hospital Essen, Essen, Germany

^5^ German Cancer Consortium (DKTK), Heidelberg, Germany

^6^ Cancer Research Center Cologne Essen (CCCE), Essen, Germany

^7^ Computational Oncology Group, Molecular Precision Oncology Program, National Center for Tumor Diseases (NCT) Heidelberg, DKFZ, Heidelberg, Germany

^8^ Pattern Recognition and Digital Medicine, Heidelberg Institute for Stem Cell Technology and Experimental Medicine (HI-STEM), Heidelberg, Germany

**SUPPLEMENTARY MATERIAL**

**Supplementary Figures**

**
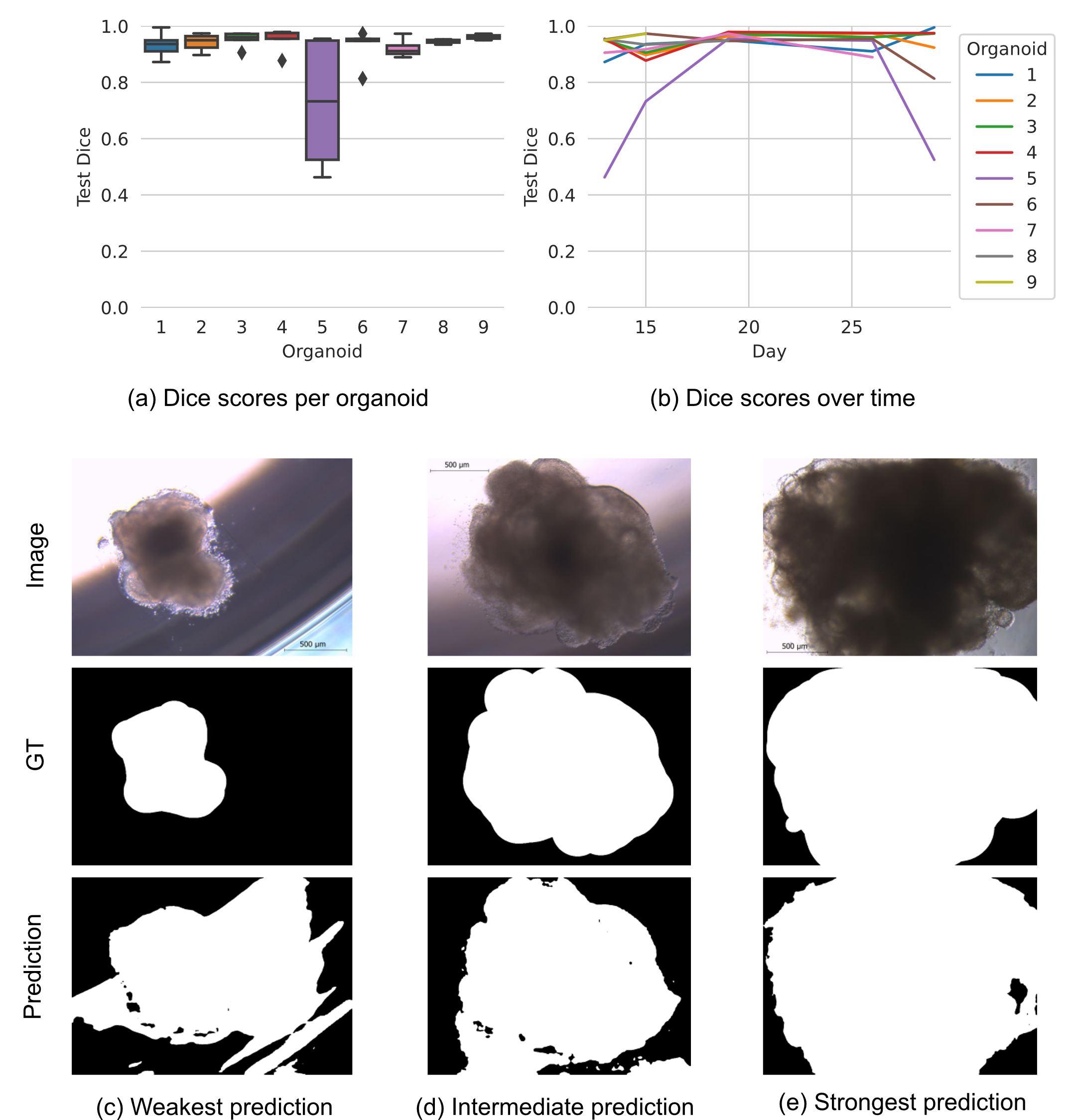
**

**Supplementary Figure 1:** **2D** **organoid segmentation for brightfield imaging**. (a) - (b) Model performance. (c) - (e) Selected organoids based on model’s prediction performance. (c) Organoid 5 (day 13): Dice score of 0.46. (d) Organoid 7 (day 15): Dice score of 0.92. (e) Organoid 4 (day 19): Dice score of 0.98. Image: original image, GT: Ground truth organoid location, Prediction: Predicted organoid location.


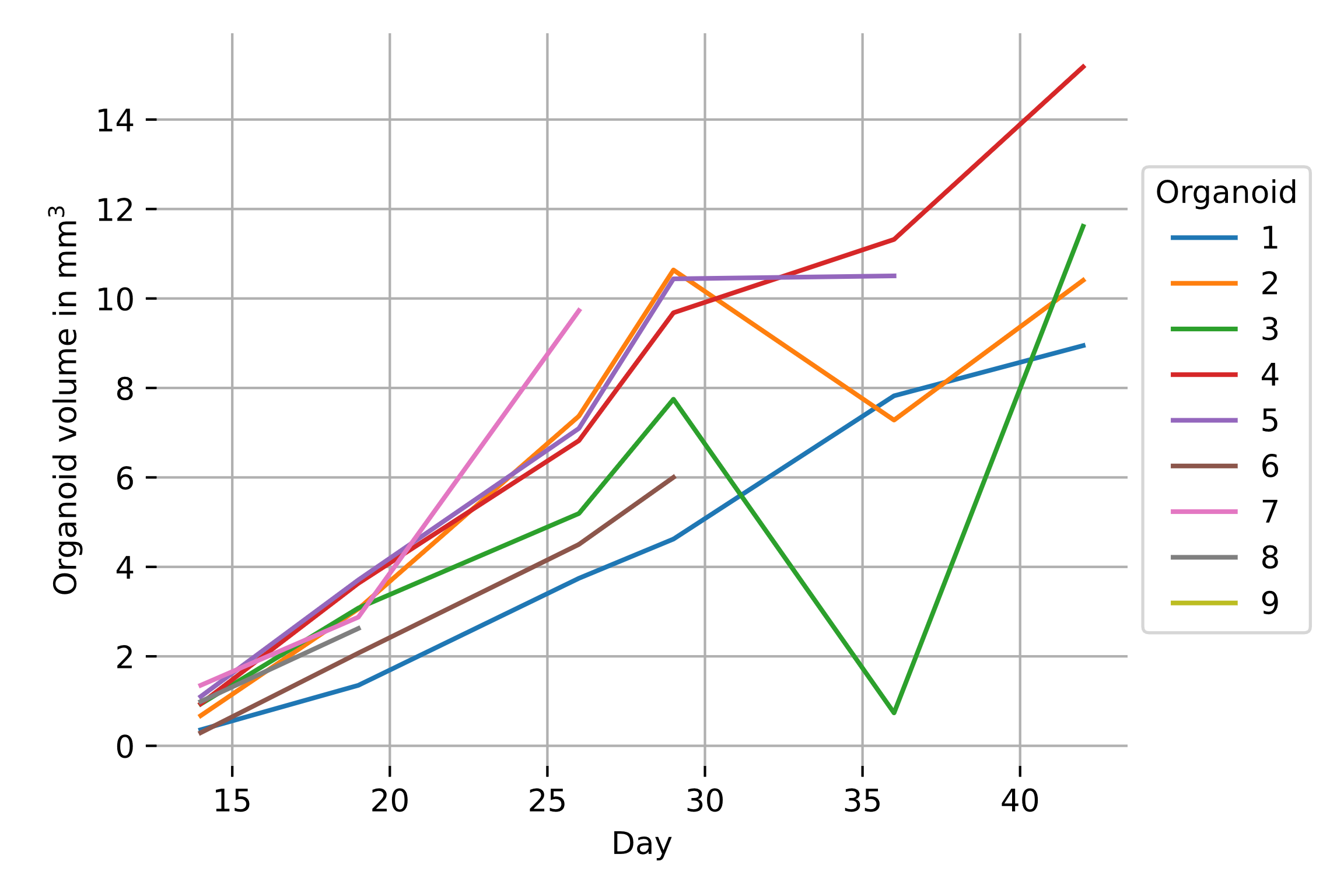


**Supplementary Figure 2:** **Organoid growth over time.** The organoid volume is based on the ground truth organoid annotation in the T2*-w sequence. Organoid 3 (day 36) has a sudden drop in volume which is due to the disruption of one or more cystic structures. Exemplary planes of this organoid are shown and discussed in the main text.

**
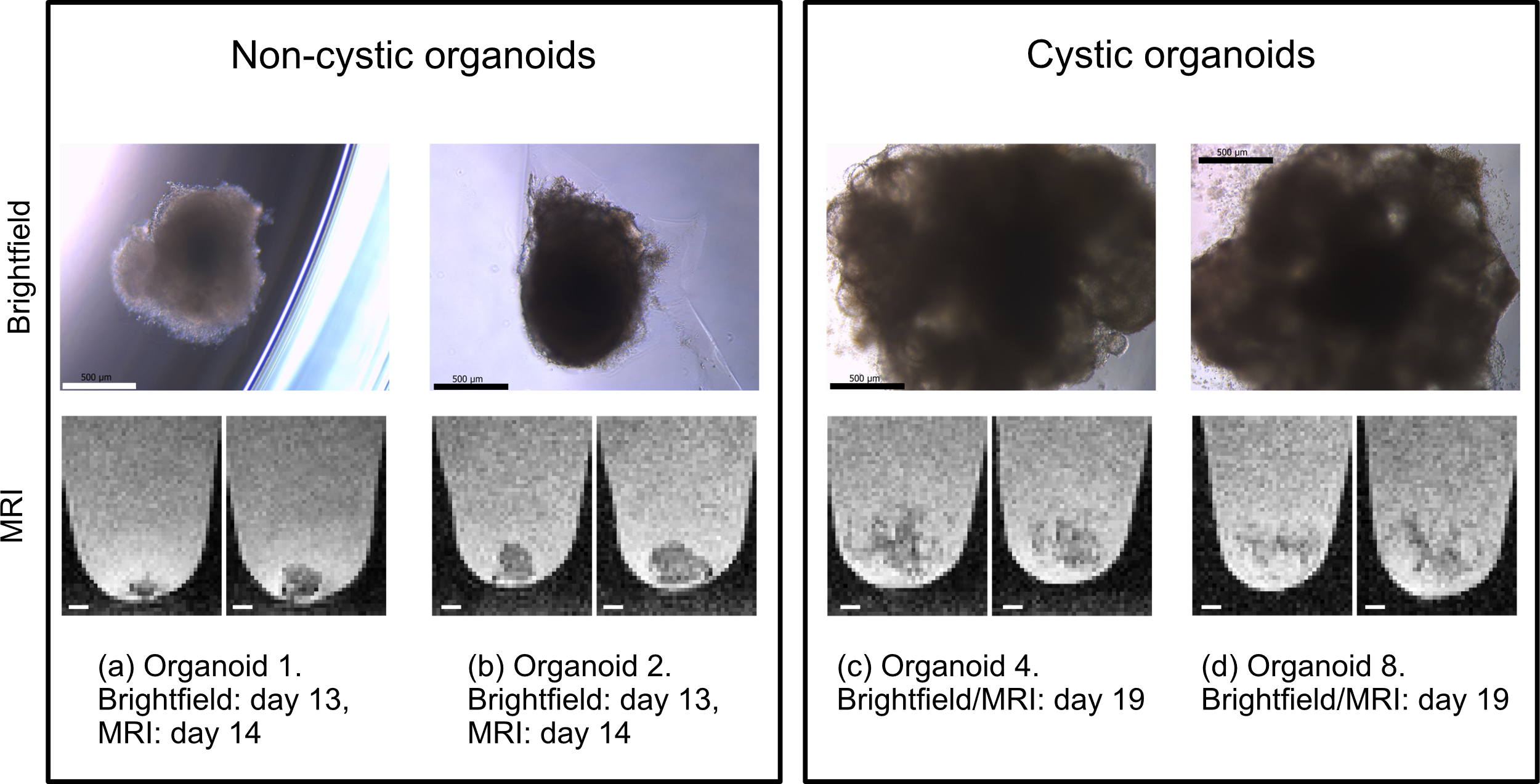
**

**Supplementary Figure 3:** **Brightfield and MRI images of two non-cystic and two cystic organoids.** The cystic organoids show fluid-filled cavities (or “cysts”) and therefore resemble the same phenotype as reported in [8, 9]. Scale bars: 500 µm (brightfield), 400 µm (MRI).

**
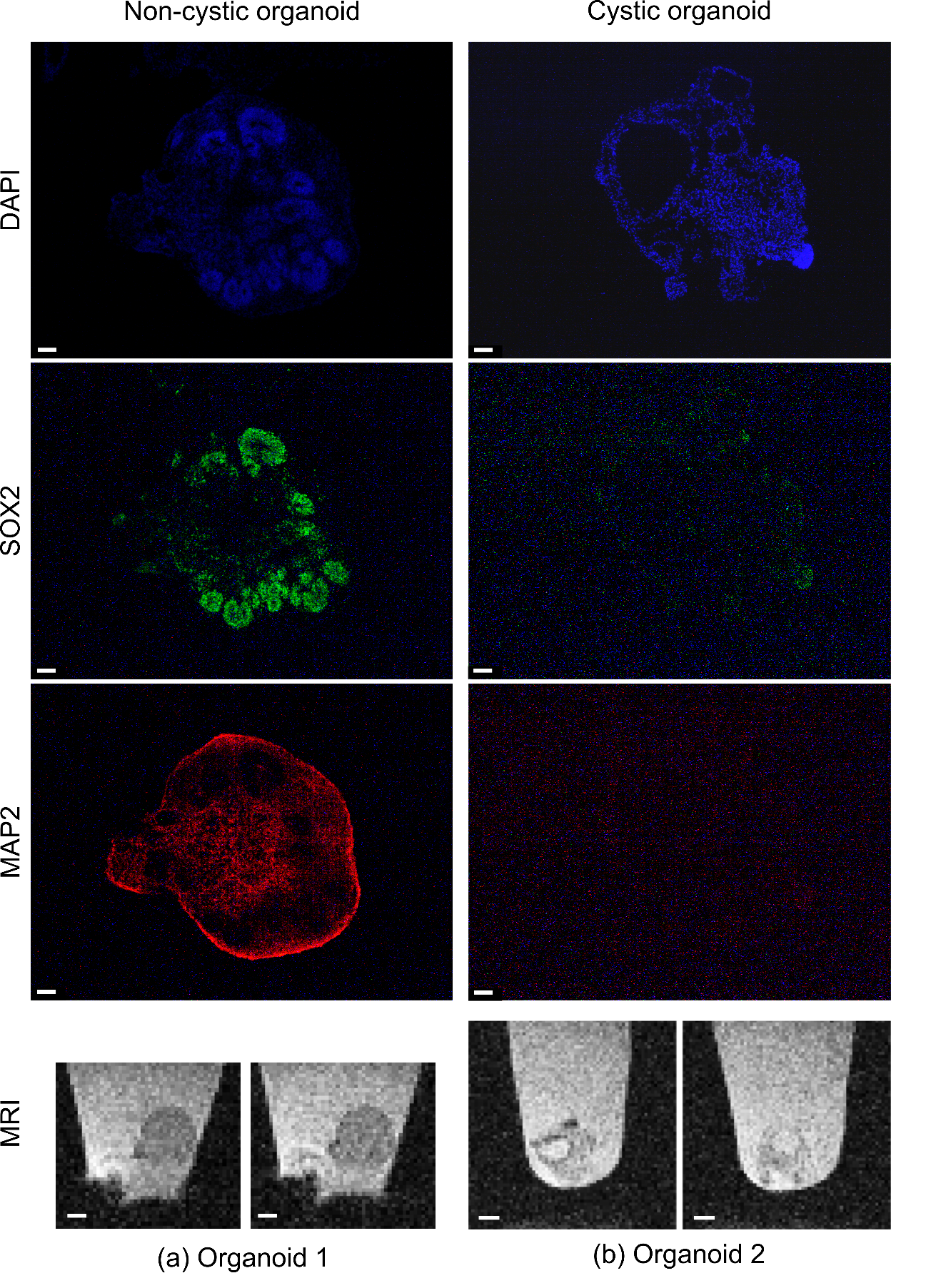
**

**Supplementary Figure 4:** **Immunofluorescence stainings and MRI images of non-cystic and cystic organoid from an extended dataset.** The non-cystic organoid grows more compact and shows ventricular-like structures containing Sox2^+^ progenitors and surrounding MAP2^+^ neurons while the cystic organoid only shows fluid-filled cavities (or “cysts”) and no generation of neural progenitors and neurons. These organoids are from an extended dataset. For better visibility, the brightness and contrast of the immunofluorescence images were enhanced. Scale bars: 50 µm (immunofluorescence images), 400 µm (MRI). Details to immunofluorescence staining and imaging are provided in the Supplementary Methods and Supplementary Table 6.

**
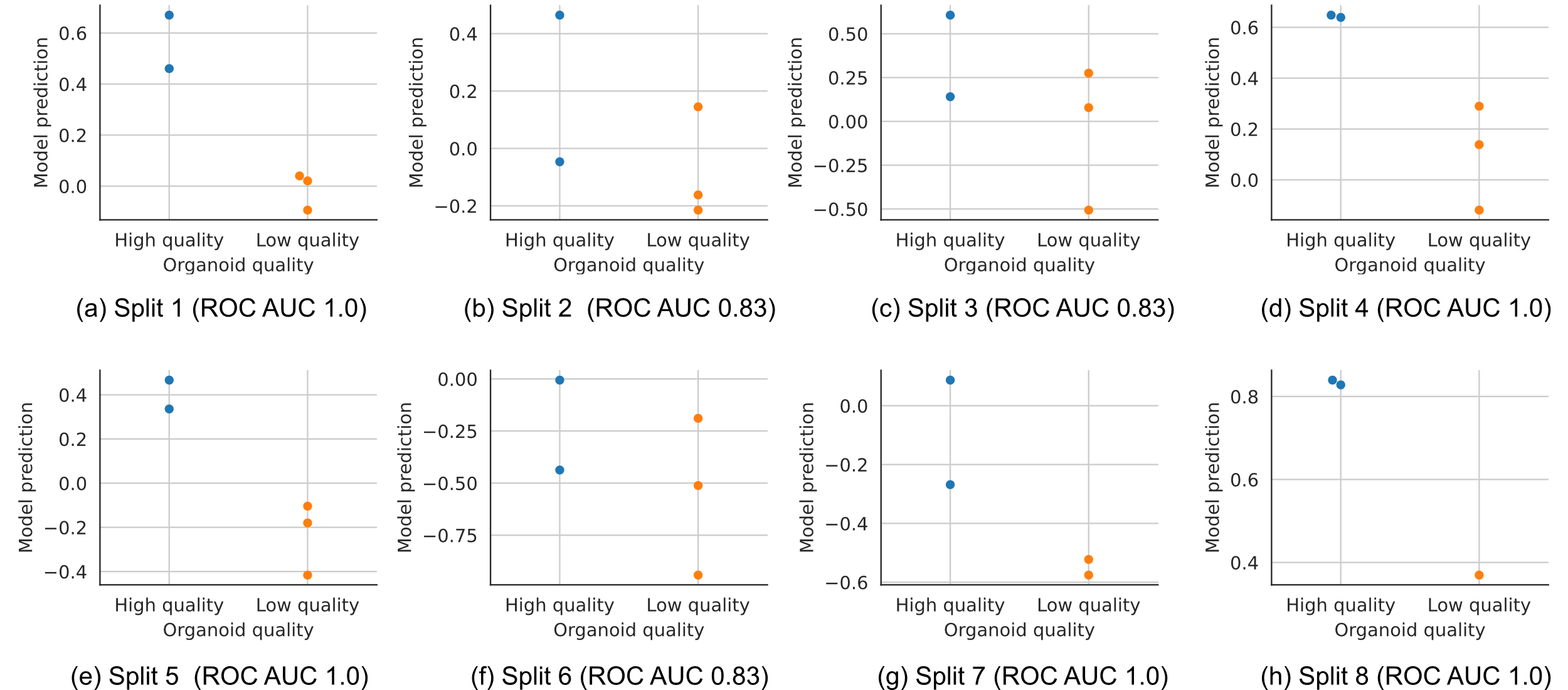
**

**Supplementary Figure 5:** **Global cysticity classification for brightfield imaging**. Each panel shows the ResNet34 test predictions for each LOOCV split. ROC AUC (mean ± SD): 0.94±0.08.


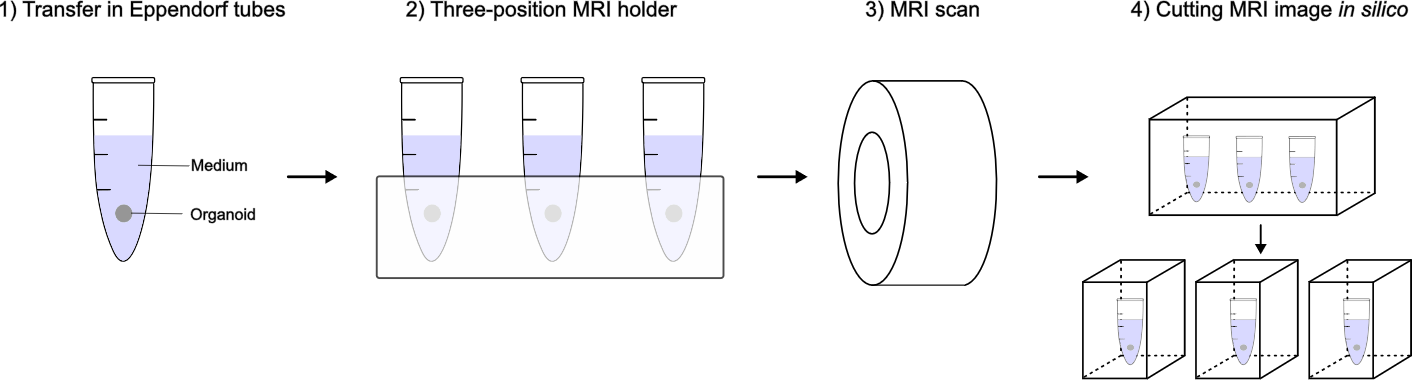


**Supplementary Figure 6:** **Experimental setup and data acquisition.** First, the cerebral organoids were transferred in medium-containing Eppendorf tubes. For MRI, three Eppendorf tubes were placed next to each other in a holder. After MRI, the MRI images were cut in silico into three equally sized image to derive one image per organoid which is essential for further automated analysis.

**
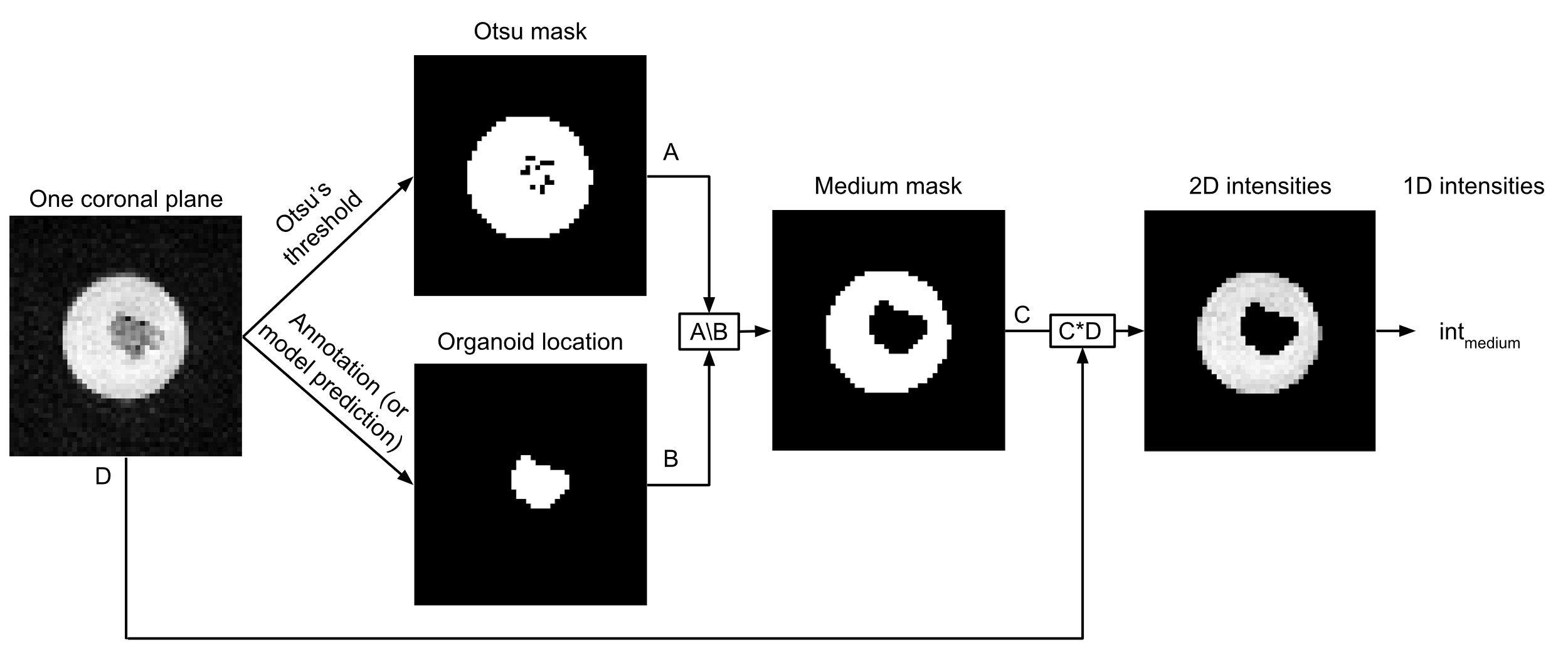
**

**Supplementary Figure 7:** **Concept of medium intensity determination for global cysticity classification in MRI.** Otsu’s mask, organoid location and medium mask are binary masks. The white pixels of the medium mask belong to the medium. This example is based on Organoid 1 (day 14), coronal plane 60. To determine the medium intensities for one organoid, this procedure is applied to all organoid-containing coronal planes from the 3D image. For better visibility in this figure, we cut the coronal plane to the Eppendorf tube boundaries.

**Supplementary Tables**

**Supplementary Table 1** ROC AUCs and adjusted p-values for separation of cystic and non-cystic organoids for selected DTI parameter maps.

| DTI parameter map | ROC AUC | P-value |
| --- | --- | --- |
| Trace | 0.91 | 1.1 × 10^-5^ |
| 3rd Eigenvalue | 0.86 | 6.2 × 10^-4^ |
| 2nd Eigenvalue | 0.91 | 1.2 × 10^-5^ |
| 1st Eigenvalue | 0.93 | 2.1 × 10^-5^ |
| Fractional Anisotropy (FA) | 0.63 | 9.9 × 10^-1^ |

**Supplementary Table 2** Organoid segmentation performance of Multi-Otsu’s threshold [30] and 2D U-Net [31] in the T2*-w sequence. Multi-Otsu’s threshold was applied in 3D for the three classes MRI background, Eppendorf tube, and organoid using the Python package scikit-image. For the 2D U-Net, the images were extracted along the coronal axis. For 2D U-Net training and evaluation, the implementation from https://github.com/milesial/Pytorch-UNet was utilized. 2D U-Net: binary semantic segmentation; 200 epochs; batch size 1; learning rate 0.00001; loss: binary cross entropy + Dice loss (weighted 1:10), weight decay: 0.001; augmentation: random rotation (probability 0.75).

| Method | Dice score (mean ± SD) |
| --- | --- |
| Multi-Otsu’s threshold | 0.08±0.09 |
| 2D U-Net | 0.58±0.43 |

**Supplementary Table 3** Blood gas analysis shows no specific negative effect of MRI on organoids. Median differences of all pre- and post-MRI measurements for medium control w/o organoid (’Medium’), MRI organoids (Org_MRI_), and control organoids (Org_control_). Cells are colored according to measurement increase or decrease.

| Measurement | Medium | Org_MRI_ | Org_control_ |
| --- | --- | --- | --- |
| pH Medium | 0.02 | -0.29 | -0.31 |
| pCO2 [mmHg] | -0.65 | 14.30 | 15.70 |
| pO2 [mmHg] | -4.10 | 2.30 | -6.40 |
| HCO3- act [mmol/l] | -0.35 | -2.60 | -2.50 |
| HCO3- std [mmol/l] | 0.50 | -7.20 | -7.50 |
| Glucose [mg/dl] | -7.00 | -22.00 | -24.00 |
| Na+ [mmol/l] | 0.20 | 1.10 | 1.70 |
| K+ [mmol/l] | 0.00 | 0.02 | 0.04 |
| Ca2+ [mmol/l] | -0.01 | 0.00 | -0.01 |
| Cl- [mmol/l] | 0.00 | 1.00 | 1.00 |

**Supplementary Table 4** Efficient 3D U-Net training and inference for organoid segmentation. For application to larger-scale experiments, it is important that the model training and especially inference time are in a practical range. The 3D U-Net requires less than an hour for training on MRI organoid segmentation using the T2*-w sequence. Inferring the model predictions is in the range of two seconds per sample. The times were measured using one NVIDIA GeForce RTX 3090 (24 GB) graphics card.

| Model | Training time (s) | | Inference time (s) | |
| --- | --- | --- | --- | --- |
|  | Per iteration | Total | Per sample | Total |
| 3D U-Net | 1.11 | 2,220 | 1.97 | 88.6 |

**Supplementary Table 5** Organoid segmentation performance of Otsu’s threshold [33] for brightfield imaging.

| Method | Dice score (mean ± SD) |
| --- | --- |
| Otsu’s threshold | 0.84±0.18 |

**Supplementary Table 6** Antibodies for immunofluorescence staining.

|  | **Antibody** | **Dilution** | **Company Cat #** | **RRID** |
| --- | --- | --- | --- | --- |
| Primary antibody | Goat anti-Sox2  Mouse anti-Map2 | 1:500  1:1000 | R&D  Sigma | AB_355110  AB_477193 |
| Secondary antibodies | Alexa Fluor donkey anti goat 488  Alexa Fluor donkey anti mouse 555 | 1:1000  1:1000 | Thermo Fisher Scientific  Thermo Fisher Scientific | AB_2534102  AB_2536180 |

**Supplementary Methods**

**Immunofluorescence staining and imaging**

For immunofluorescence staining, organoids were fixed in 4% paraformaldehyde (PFA)/DPBS over night at 4°C. The following day, PFA solution was removed, the organoids were washed twice in DPBS and places into 30% sucrose solution until the sunk to the bottom of the tube. For cryosectioning, organoids were embedded into HistoVT (GERBU Bio) and frozen in dry ice. Organoids were cut into 20 µM sections and transferred to a slide for staining. Slides were dried overnight at room temperature. For staining, slides were rehydrated with DPBS and incubated for 15min in 0.5% Triton-X-100/DPBS for permeabilization and blocked for 1h with blocking solution (4% BSA/DPBS). Sections were then incubated with the respective primary antibodies (Supplementary Table 6) in blocking solution overnight at 4°C. The following day, slides were washed twice in 0.5% Triton-X-100/DPBS and the secondary antibodies (Supplementary Table 6) were added in blocking solution for two hours at room temperature followed by two washing steps with 0.5% Triton-X-100/DPBS. Nuclei were stained with DAPI (1µg/ml) in DPBS for 5min at room temperature and washed once with DPBS for 5min. Slides were dried at room temperature and embedded into Fluoromount G. Slides were kept at 4°C until imaging. Image acquisition was performed with a Leica DMi 4000 B fluorescence microscope using Leica imaging software.
